# Renin-Angiotensin System Inhibitor is Associated with Lower Risk of Ensuing Chronic Kidney Disease after Functional Recovery from Acute Kidney Injury

**DOI:** 10.1038/srep46518

**Published:** 2017-04-13

**Authors:** Yu-Hsiang Chou, Tao-Min Huang, Szu-Yu Pan, Chin-Hao Chang, Chun-Fu Lai, Vin-Cent Wu, Ming-Shiou Wu, Kwan-Dun Wu, Tzong-Shinn Chu, Shuei-Liong Lin

**Affiliations:** 1 Renal Division, Department of Internal Medicine, National Taiwan University Hospital, Taipei, Taiwan; 2 Department of Internal Medicine, National Taiwan University Hospital Jin-Shan Branch, New Taipei City, Taiwan; 3 Graduate Institute of Physiology, College of Medicine, National Taiwan University, Taipei, Taiwan; 4 Department of Internal Medicine, Far Eastern Memorial Hospital, New Taipei City, Taiwan; 5 Department of Medical Research, National Taiwan University Hospital, Taipei, Taiwan; 6 Department of Integrated Diagnostics &Therapeutics, National Taiwan University Hospital, Taipei, Taiwan; 7 Research Center for Developmental Biology and Regenerative Medicine, National Taiwan University, Taipei, Taiwan

## Abstract

Acute kidney injury (AKI) is an independent risk factor for ensuing chronic kidney disease (CKD). Animal studies have demonstrated that renin-angiotensin system (RAS) inhibitor can reduce ensuing CKD after functional recovery from AKI. Here we study the association between ensuing CKD and use of RAS inhibitor including angiotensin converting enzyme inhibitor or angiotensin II type 1a receptor blocker starting after renal functional recovery in our prospectively collected observational AKI cohort. Adult patients who had cardiac surgery–associated AKI (CSA-AKI) are studied. Patients with CKD, unrecovered AKI, and use of RAS inhibitor before surgery are excluded. Among 587 eligible patients, 94 patients are users of RAS inhibitor which is started and continued after complete renal recovery during median follow-up period of 2.99 years. The users of RAS inhibitor show significantly lower rate of ensuing CKD (users vs. non-users, 26.6% vs. 42.2%) and longer median CKD-free survival time (users vs. non-users, 1079 days vs. 520 days). Multivariate Cox regression analyses further demonstrate that use of RAS inhibitor is independently associated with lower risk of ensuing CKD (hazard ratio = 0.46, *P* < 0.001). We conclude that use of RAS inhibitor in CSA-AKI patients after renal functional recovery is associated with lower risk of ensuing CKD development.

Acute kidney injury (AKI) is common and associated with higher morbidity and mortality globally^1^,^2^,^3^,^4^. AKI has also been recognized as a major risk factor for the development of chronic kidney disease (CKD)^5^,^6^. Mounting evidence has shown that AKI and CKD appear to be an interconnected syndrome^7^. The severity, duration and frequency of AKI has been linked to the development and progression of ensuing CKD^6^,^7^,^8^,^9^,^10^. Continuous monitoring of renal function has been emphasized, even if patients have shown functional recovery after AKI^5^,^6^. Many studies have focused on the prevention and management of AKI to reduce the ensuing CKD development^11^,^12^,^13^. Nevertheless, more efforts are needed to develop strategies for blocking AKI-CKD transition after functional recovery.

Although the mechanisms underlying AKI-CKD transition are incompletely understood in humans, animal studies have shown a number of pathogenetic mechanisms such as maladaptive repair^14^, profibrogenic cytokine production by G2/M cell-cycle arrested epithelia^15^, pericyte-myofibroblast transition^16^,^17^, and microvascular rarefaction^18^,^19^. These mechanisms open up opportunities to innovate therapeutic strategies for prevention of AKI-CKD transition. For AKI patients with incomplete recovery of renal function, we can treat them as CKD patients. However, we have neither consensus nor reliable therapeutic interventions for patients with renal functional recovery from AKI.

Recently, animal studies have demonstrated that activation of intrarenal renin–angiotensin system (RAS) after AKI underlies the possible mechanism for development and progression of ensuing CKD^20^,^21^,^22^. To get insight into the clinical application of RAS inhibitor and its impact on development of ensuing CKD in AKI survivors with complete renal recovery, we studied the outcomes and relevant risk factors of cardiac surgery–associated AKI (CSA-AKI) patients who did not have CKD history before surgery in our prospectively collected observational cohort.

## Results

### Baseline Characteristics and Follow-up for CKD Development

**Figure 1:**
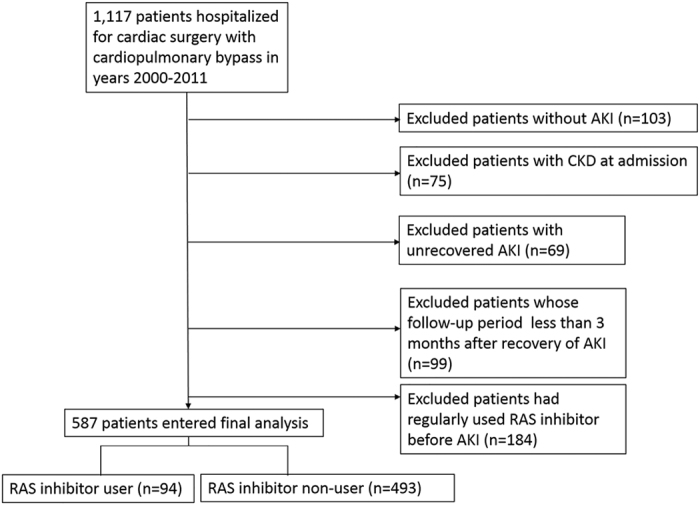
Flow diagram of patient enrollment. Patients hospitalized between January 1, 2000 and December 31, 2011 were screened using inclusion and exclusion criteria. Totally 587 patients were identified for final analysis. Abbreviation: AKI, acute kidney injury; CKD, chronic kidney disease; RAS, renin-angiotensin system.

Of the 1117 patients who underwent cardiac surgery during the period of January 1, 2000 and December 31, 2011, 530 patients were excluded from analysis due to no AKI (103 patients), diagnosis of CKD or estimated glomerular filtration rate (eGFR) <60 ml/min/1.73 m^2^ before surgery (75 patients), unrecovered AKI (69 patients), follow-up less than 3 months after recovery from AKI (99 patients) and regular medication with RAS inhibitor including angiotensin converting enzyme (ACE) inhibitor or angiotensin II type 1a receptor blocker (ARB) before AKI (184 patients). Of the 587 eligible patients, 94 patients were users of RAS inhibitor which was started after complete renal recovery defined as the decrease of serum creatinine (SCr) level to within 0.3 mg/dl above the baseline and continued during the median follow-up period of 2.99 years (Fig. 1). The other 493 patients were non-users of RAS inhibitor.

The clinical characteristics of the study population were shown in Table 1. Mean age of the patients was 61.8 years old and 69.2% were male. Most patients experienced stage 1 AKI (91.8%). Nearly 40% patients received coronary artery bypass grafting (CABG). Coronary artery disease (CAD, 62.4%) and hypertension (HTN, 54.0%) were the two major comorbidities. HTN was noted in 93.6% and 46.5% of users and non-users of RAS inhibitor, respectively (*P* < 0.001). At discharge from hospital, 87.2% of users and 26.8% of non-users had treatment with anti-HTN agents (*P* < 0.001). In contrast, more non-users of RAS inhibitor had congestive heart failure (CHF), metastatic cancer and immunosuppressant treatment.

In the follow-up period after complete renal recovery from AKI, there was no significant difference of SCr at AKI recovery between two groups (Table 1). Of all patients, 39.7% developed CKD which was determined by eGFR <60 ml/min/1.73 m^2^ (Table 2). In users of RAS inhibitor, 26.6% developed CKD, which was much less than 42.2% in non-users (*P* = 0.005). The median CKD-free survival time 1079 days in users was much longer than 520 days in non-users (*P* = 0.011).

### Cox Regression Analyses of Risk Factors for CKD Development

**Figure 2:**
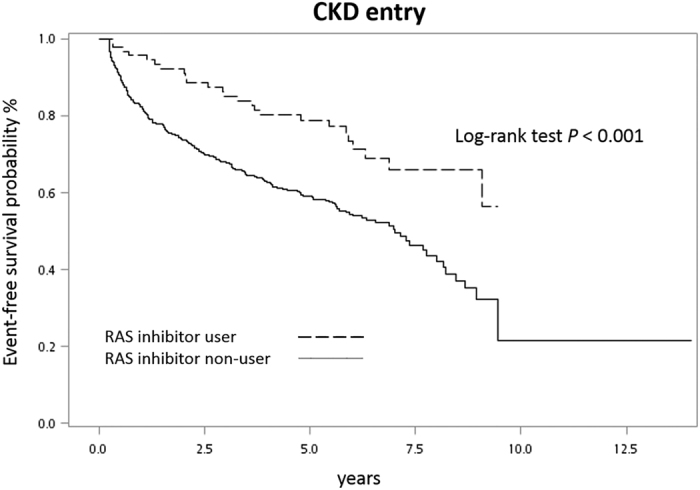
Kaplan-Meier analysis of CKD-free-survival for users and non-users of RAS inhibitor.

We then performed univariate and multivariate Cox regression analyses to identify independent factors for ensuing CKD development (Table 3). Notably, use of RAS inhibitor was independently associated with lower risk (hazard ratio [HR] = 0.46, *P* < 0.001). The other factors significantly protective against ensuing CKD were higher levels of baseline hemoglobin and eGFR. Conversely, variables significantly associated with risk of ensuing CKD included older patients, higher SCr level at AKI, diabetes mellitus (DM), CHF, HTN and hyperuricemia. The Kaplan-Meier curve revealed significant protection of RAS inhibitor from ensuing CKD again (Fig. 2).

**Figure 3:**
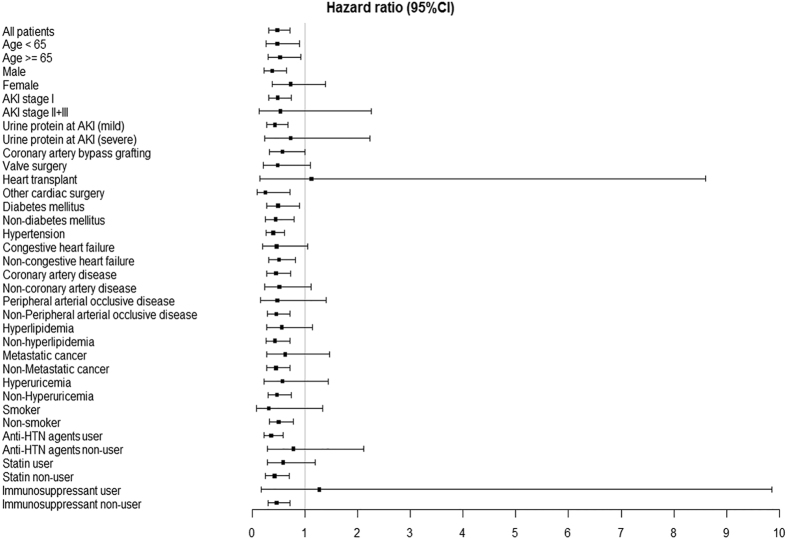
Hazard ratio (95% confidence interval) for ensuing CKD associated with use of RAS inhibitor in subgroups of enrolled patients. Abbreviation: CI, confidence interval; HTN, hypertension.

We further performed subgroup analyses (Fig. 3). Use of RAS inhibitor was still significantly associated with lower risk of ensuing CKD in most subgroups except female patients, patients with AKI stage II/III, severe proteinuria at AKI, peripheral arterial occlusive disease (PAOD), hyperlipidemia, hyperuricemia, metastatic cancer, non-users of anti-HTN agents, users of statins or immunosuppressants, smokers and heart transplant recipients. RAS inhibitor had borderline protective effect on subgroups of patients undergoing CABG or valve surgery as well as patients with CHF and non-coronary artery disease. On the other hand, the HR of ensuing CKD was much lower in several subgroups, including male patients, other cardiac surgery and anti-HTN agent users.

## Discussion

There is substantial progress in the field of AKI over the past 10 years^23^,^24^. The previous conventional wisdom that AKI survivors with complete renal recovery tend to enjoy good health appears to be flawed^6^,^25^,^26^,^27^,^28^. Most of the previous studies had various definition of renal recovery, including weaning from dialysis, return of eGFR to >90% of reference or return of SCr to within 20% of baseline, etc.^29^,^30^. Although the Acute Dialysis Quality Initiative (ADQI) consensus defines complete renal recovery as return to baseline classification within the RIFLE criteria and partial recovery as a change in RIFLE status in patient free of dialysis^31^, few studies have evaluated renal recovery in accordance with this recommendation. Even KDOQI guideline has no definition of complete renal recovery. The lack of a consistent definition for renal recovery is an obstacle for adequate comparison between studies for incidence of subsequent CKD and to develop strategies for patient monitoring and treatment after AKI. Therefore, renal recovery defined as the decrease of SCr level to within 0.3 mg/dl above the baseline in our patients without CKD history before AKI is probably most close to concept of complete renal recovery in ADQI consensus. Moreover, it is important to clarify the outcome of these patients with complete renal recovery, who are thought to do well and lack of continuous monitoring of renal function in general. After AKI, we can treat patients without complete renal recovery as CKD, but we may miss the golden time to prevent the development and progression of ensuing CKD in patients with complete renal recovery if we pursue the conventional wisdom.

Our results confirmed that 39.7% of CSA-AKI survivors with complete renal recovery developed CKD during median follow-up duration of 2.99 years. Use of RAS inhibitor after complete renal recovery from CSA-AKI was strikingly associated with risk reduction by 54% for ensuing CKD development after multivariate adjustment. To our knowledge, this is the first clinical report to confirm the association of RAS inhibitor with the risk reduction for ensuing CKD in patients with complete renal recovery from AKI. Moreover, this clinical study confirms our previous study in mice that RAS inhibition by losartan can reduce ensuing CKD and mortality after functional recovery from AKI induced by ischemia-reperfusion injury^20^. To minimize the impact of different etiologies of AKI on ensuing CKD development and on the protective effect of RAS inhibitor, we chose patients undergoing cardiac surgery with cardiopulmonary bypass (CPB) as the study population from our prospectively collected observational cohort^6^,^13^,^28^,^32^,^33^,^34^,^35^,^36^. CSA-AKI is the second most common cause of AKI in the intensive care unit^37^. Ischemia-reperfusion injury, similar to the mechanism responsible for the animal model used in our recent study regarding RAS inhibition on AKI-CKD transition^20^, is thought to play a major role in the pathogenesis of CSA-AKI^38^,^39^. The striking and consistent effect on risk reduction for ensuing CKD development in clinical cohort and mouse model of AKI-CKD continuum provides evidence for clinical application of RAS inhibitor to prevent ensuing CKD development in AKI survivors with complete renal recovery even patients do not have CKD history before AKI.

Burgeoning studies have shown that mild AKI can increase the risk of CKD and mortality and postulated a persistent pathophysiological change in kidney even patients achieve functional recovery^6^,^40^. Several mechanisms responsible for the AKI-CKD transition have been demonstrated. These mechanisms include tubular cell loss^41^, tubular cell G2/M cell-cycle arrest^15^, persistent inflammation^42^, microvascular rarefaction^18^,^19^, and epigenetic change related cell proliferation of pericytes/fibroblasts after AKI^17^,^43^,^44^. Moreover, abnormal renal pathology and ongoing injury are still noted in a murine model even biochemical parameters of renal function have returned to baseline after AKI^20^. Our previous study has shown the upregulation of genes *Agt* and *Agtr1a* that encoded angiotensinogen and angiotensin II type 1a receptor respectively in injured kidneys, suggesting ongoing activation of intrarenal RAS^20^. It is noteworthy that some studies indicate the activation of RAS after AKI. In CSA-AKI, low cardiac output before, during, or after surgery is directly related to AKI risk due to increased renal vasoconstriction via RAS activation^45^. In addition, overexpression of intrarenal RAS is reported in patients with acute tubular necrosis and is associated with the severity of AKI and urinary levels of angiotensinogen reflect intrarenal RAS activity^46^,^47^,^48^. Incomplete tubular epithelial regeneration results in nephron loss and hyperfiltration in the remaining glomeruli^49^. RAS activation is the plausible cause for this change to maintain glomerular filtration after AKI. This mechanism appears to be one of the mechanisms for the elevated blood pressure after AKI in a recent clinical study as well^50^. Many clinical trials have proved the specific renoprotective effect of RAS inhibition by ACE inhibitor/ARB for patients with diabetic or proteinuric non-diabetic CKD to reduce disease progression and mortality^51^,^52^,^53^. However, RAS inhibition is usually avoided during the acute phase of AKI patients, and the role of RAS activity in acute phase and injury severity is not clear indeed^21^,^22^,^54^. Based on findings that intrarenal RAS was activated in repairing kidneys in spite of complete recovery of plasma parameters for renal function assessment one month after acute injury, our previous study has shown that RAS inhibition with losartan in mouse AKI survivors can prevent the development of ensuing CKD and mortality^20^. Furthermore, focal tubular atrophy, ongoing inflammation, and intrarenal RAS activation led to a vicious cycle in repairing kidneys for ensuing CKD progression even plasma biochemical parameters showed recovery from AKI. Evidence becomes more clear that RAS inhibitor can provide a key to break the vicious cycle for AKI-CKD transition.

Moreover, RAS inhibitors may prevent ensuing CKD indirectly thorough reduction of cardiorenal syndrome^55^. RAS inhibitor therapy has been included in major society guideline of heart failure management^56^, because multiple clinical trials have shown that RAS inhibitor therapy leads to symptomatic improvement, reduced hospitalization, and lower mortality in patients with heart failure^57^. This cardiovascular protective effect could reduce acute or chronic cardiorenal syndrome related ensuing CKD.

Our analyses confirmed that traditional risk factors for CKD development^58^,^59^, such as old age, DM, higher SCr at AKI, HTN, CHF and hyperuricemia have significant impact for AKI-CKD transition. Our analyses also showed that higher hemoglobin and baseline eGFR can protect from ensuing CKD, possibly through reducing hypoxia during AKI and higher renal reserve as reported previously^60^,^61^.

Our subgroup analyses showed that groups of male patients, other cardiac surgery and anti-HTN agents acquire a higher magnitude of benefits from RAS inhibitor than all patients, suggesting higher RAS activity in these subgroups. On the contrary, some subgroups had no significant risk reduction of CKD development even under RAS inhibition. In addition to RAS activation, another dominant mechanism need to be unraveled for AKI-CKD transition in groups of female patients, severe AKI (stage II/III), severe proteinuria at AKI, PAOD, hyperuricemia, hyperlipidemia, smokers, metastatic cancer and non-users of anti-HTN agents. No benefits for heart transplant recipients and statins or immunosuppressant users could be due to different pathogenesis of its correspondingly underlying disease or its specific drug effect.

There are some limitations in our study. First, this was an observational study. Well designed-clinical trials should be initiated in the future to prove the protective effect of RAS inhibitor on ensuing CKD after complete renal recovery from AKI. Second, 10.2% of patients without CKD development received follow-up period less than one year which may underestimate the incidence of ensuing CKD after AKI. It might take longer time for our patients to develop CKD because most patients had mild AKI and median duration required for ensuing CKD development was far more than one year. Third, only patients with CSA-AKI were included. To extend our findings, we need more studies to investigate the effect of RAS inhibitor after complete renal recovery from AKI due to the other mechanisms. Finally, urinalysis was not checked as frequently as SCr. Therefore, any abnormal findings in urine representing the residual renal damage in patients could not be timely noticed. Even though these limitations, this prospectively collected observational cohort study provided the strong evidence that use of RAS inhibitor after complete renal recovery from CSA-AKI was associated with risk reduction by 54% for ensuing CKD after multivariate adjustment.

In conclusion, in patients without CKD history, use of ACE inhibitor or ARB was associated with lower risk of ensuing CKD development after complete renal recovery from CSA-AKI. Our study highlights the important role of RAS activation in AKI-CKD transition. Use of RAS inhibitor should be included in the strategies for post-AKI care.

## Methods

### Patients

This was a prospectively collected observational cohort study based on the National Taiwan University Hospital Study Group on Acute Renal Failure (NSARF) database established in the surgical intensive care units (SICU). We screened patients in this database who were ≥18 years old and hospitalized in the SICU of National Taiwan University Hospital (NTUH) for postoperative care of cardiac surgery with CPB between January 1, 2000 and December 31, 2011. We excluded patients who did not have AKI after surgery. Other exclusion criteria were history of CKD, preoperative eGFR <60 ml/min/1.73 m^2^ by Taiwanese MDRD equation^62^, no recovery of SCr to level within 0.3 mg/dl above baseline within one month after AKI, follow-up period less than 3 months after AKI, and medication with RAS inhibitor including ACE inhibitor or ARB before AKI. The follow-up was continued until September 30, 2016.

The study was approved by the Institutional Review Board of NTUH. A waiver of informed consent was obtained because there was no breach of privacy and no interference with patient care.

### Clinical Assessment of Patients

Preoperative demographic data were obtained at SICU admission. These variables included age, gender, smoking history, DM (using oral hypoglycemic agents or insulin), HTN (using anti-HTN agents or systolic/diastolic blood pressure >140/90 mmHg at admission), CHF (defined as New York Heart Association (NYHA) functional class III or IV), PAOD (defined by clinical or imaging diagnosis), chronic obstructive pulmonary disease (with long-term bronchodilators), hyperlipidemia (with lipid-lowering agents) and CAD (defined by the diagnostic code of ischemic heart disease prior to admission, and positive electrocardiographic findings). Chronic hepatitis and metastatic cancer were also recorded according to diagnostic codes prior to admission.

Laboratory data such as baseline SCr, albumin, hemoglobin were recorded at SICU admission. SCr at the peak of AKI was also obtained. Urine protein at AKI was recorded according to urine dipstick test. Mild proteinuria and severe proteinuria were defined by the result of trace to 1 + and 2 + to 4 + respectively. Baseline eGFR was calculated using Taiwanese MDRD equation^62^. The surgical procedure was categorized into CABG, heart valve surgery, heart transplant and others. The AKI definition and staging were based on Kidney Disease: Improving Global Outcomes (KDIGO) criteria^63^.

Medications such as anti-HTN agents (including calcium channel blockers, β-blockers, α-blockers, clonidine), lipid-lowering agents (statins), immunosuppressants after heart transplant were recorded during hospitalization and at discharge. Users of RAS inhibitor were defined as starting ACE inhibitor or ARB between 1 and 6 months after complete renal recovery from AKI and continuing the medication during follow-up, while the others were defined as non-users.

In addition to data collection during hospitalization and at discharge, we also leveraged an electric medical record to keep track of important covariates when patient visited our outpatient department, including SCr, medications and diagnosis of comorbidity.

### Outcome

The endpoint was stage 3 CKD development during the follow-up period. All of the longitudinal measurements of SCr and eGFR during hospitalization and follow-up period were obtained for each enrolled patient. Stage 3 CKD was determined by eGFR below 60 ml/min/1.73 m^2 ^62. Patient who lost follow-up before September 30, 2016 would be seen as censored data.

### Statistical Analysis

We performed statistical analyses with the SAS software, version 9.4. Continuous variables were presented as mean (standard deviation, SD), and the difference between users and non-users of RAS inhibitor was compared with the Student’s t-test. Categorical variables were summarized as percentages and analyzed with the chi-square test. Two-sided *P* < 0.05 was considered statistically significant. We constructed a univariate and multivariate Cox regression model to investigate the association between use of RAS inhibitor and CKD development. Variables significantly associated with CKD development in the univariate analysis (*P* < 0.05) were included in the multivariate Cox regression model. The survival curves for CKD development were plotted using Kaplan-Meier method. Subgroup analyses were also performed to estimate the HR. Subgroups of variables deemed clinical relevant to CKD development, which included age, sex, DM, HTN, CHF, CAD, PAOD, hyperlipidemia, hyperuricemia, metastatic cancer, smoking status, AKI stage, urine protein at AKI, surgical procedures, medication use such as anti-HTN agents, statins or immunosuppressants were identified.

## Additional Information

**How to cite this article:** Chou, Y.-H. *et al*. Renin-Angiotensin System Inhibitor is Associated with Lower Risk of Ensuing Chronic Kidney Disease after Functional Recovery from Acute Kidney Injury. *Sci. Rep.* **7**, 46518; doi: 10.1038/srep46518 (2017).

**Publisher's note:** Springer Nature remains neutral with regard to jurisdictional claims in published maps and institutional affiliations.

## Figures and Tables

**Table 1:** Baseline clinical characteristics of patients.

	Overall (*n* = 587)	Users of RAS inhibitor (*n* = 94)	Non-users of RAS inhibitor (*n* = 493)	*P* value
Demographic characteristics				
Age, years	61.8 (14.8)	60.9 (12.3)	62.0 (15.2)	0.45
Man, *N* (%)	406 (69.2%)	69 (73.4%)	337 (68.4%)	0.33
Diabetes mellitus, *N* (%)	184 (31.4%)	31 (33.0%)	153 (31.0%)	0.71
Hypertension, *N* (%)	317 (54.0%)	88 (93.6%)	229 (46.5%)	< 0.001
Congestive heart failure NYHA III or IV, *N* (%)	191 (32.5%)	21 (22.3%)	170 (34.5%)	0.02
Coronary artery disease, *N* (%)	366 (62.4%)	66 (70.2%)	300 (60.9%)	0.09
Peripheral arterial occlusive disease, *N* (%)	54 (9.2%)	10 (10.6%)	44 (8.9%)	0.60
Hyperlipidemia, *N* (%)	163 (27.8%)	28 (29.8%)	135 (27.4%)	0.63
Chronic obstructive pulmonary disease, *N* (%)	70 (11.9%)	11 (11.7%)	59 (12.0%)	0.94
Chronic hepatitis, *N* (%)	16 (2.7%)	1 (1.1%)	15 (3.0%)	0.28
Hyperuricemia, *N* (%)	81 (13.8%)	10 (10.6%)	71 (14.4%)	0.33
Metastatic cancer, *N* (%)	140 (23.9%)	19 (20.2%)	121 (24.5%)	0.37
Current or former smoker, *N* (%)	50 (8.5%)	11 (11.7%)	39 (7.9%)	0.23
Laboratory data				
Baseline hemoglobin, g/dl (SD)	13.2 (1.8)	13.5 (1.6)	13.2 (1.8)	0.09
Baseline albumin, g/dl (SD)	4.2 (0.5)	4.3 (0.5)	4.2 (0.5)	0.55
Baseline SCr, mg/dl (SD)	0.85 (0.18)	0.87 (0.16)	0.85 (0.19)	0.28
Baseline eGFR, ml/min/1.73 m^2^ (SD)	89.0 (25.2)	86.3 (18.4)	89.5 (26.3)	0.15
SCr at AKI, mg/dl (SD)	1.65 (1.01)	1.61 (0.85)	1.66 (1.04)	0.60
SCr at AKI recovery, mg/dl (SD)	0.91 (0.06)	0.94 (0.17)	0.91 (0.08)	0.14
Urine protein at AKI (severe), *N* (%)	28 (4.8%)	7 (7.5%)	21 (4.3%)	0.18
AKI stage, *N* (%)				
Stage I	539 (91.8%)	89 (94.7%)	450 (91.3%)	0.37
Stage II + Stage III	48 (8.2%)	5 (5.3%)	43 (8.7%)	0.37
Surgical procedure, *N* (%)				
Coronary artery bypass grafting	232 (39.5%)	38 (40.4%)	194 (39.4%)	0.94
Valve surgery	217 (37.0%)	28 (29.8%)	189 (38.3%)	0.15
Heart transplant	37 (6.3%)	2 (2.1%)	35 (7.1%)	0.11
Other cardiac surgery	101 (17.2%)	26 (27.7%)	75 (15.2%)	0.005
Medication at discharge, *N* (%)				
Anti-HTN agents	214 (36.5%)	82 (87.2%)	132 (26.8%)	<0.001
Statins	149 (25.4%)	28 (29.8%)	121 (24.5%)	0.28
Immunosuppressants	37 (6.3%)	2 (2.1%)	35 (7.1%)	0.07

Abbreviation: AKI, acute kidney injury; eGFR, estimated glomerular filtration rate; HTN, hypertension; NYHA, New York Heart Association; RAS, renin-angiotensin system; SCr, serum creatinine.

**Table 2:** Development of chronic kidney disease during follow-up.

	Overall (*n* = 587)	Users of RAS inhibitor (*n* = 94)	Non-users of RAS inhibitor (*n* = 493)	*P* value
CKD development, *N* (%)	233 (39.7%)	25 (26.6%)	208 (42.2%)	0.005
Median CKD-free survival time, days	574	1079	520	0.011

Abbreviation: CKD, chronic kidney disease.

**Table 3:** Cox regression analyses for independent factors associated with CKD development.

Covariate	Univariate Analysis		Multivariate analysis	
	HR (95% CI)	*P*	HR (95% CI)	*P*
Demographic characteristics				
Age	1.04 (1.03–1.05)	<0.001	1.03 (1.02–1.05)	<0.001
Sex^a^	0.81 (0.62–1.06)	0.12		
Diabetes mellitus^b^	1.99 (1.53–2.57)	<0.001	1.61 (1.23–2.10)	0.001
Hypertension^b^	1.56 (1.20–2.02)	0.001	1.48 (1.12–1.95)	0.006
Congestive heart failure NYHA III or IV^b^	1.38 (1.06–1.81)	0.02	1.38 (1.05–1.81)	0.02
Coronary artery disease^b^	1.23 (0.93–1.61)	0.15		
Peripheral arterial occlusive disease^b^	1.44 (0.94–2.21)	0.09		
Hyperlipidemia^b^	0.99 (0.75–1.31)	0.95		
Chronic obstructive pulmonary disease^b^	1.01 (0.67–1.52)	0.96		
Chronic hepatitis^b^	1.78 (0.88–3.60)	0.11		
Hyperuricemia^b^	1.97 (1.44–2.70)	<0.001	1.64 (1.19–2.27)	0.003
Metastatic cancer^b^	0.96 (0.71–1.30)	0.79		
Current or former smoker^b^	0.97 (0.61–1.55)	0.90		
Laboratory data				
Baseline hemoglobin, g/dl	0.86 (0.80–0.93)	<0.001	0.91 (0.84–0.99)	0.02
Baseline albumin, g/dl	0.68 (0.52–0.89)	0.005	1.17 (0.86–1.59)	0.32
Baseline SCr, mg/dl	8.72 (4.09–18.58)	<0.001		
Baseline eGFR, ml/min/1.73 m^2^	0.96 (0.96–0.97)	<0.001	0.98 (0.97–0.99)	<0.001
SCr at AKI, mg/dl	1.47 (1.35–1.60)	<0.001	1.52 (1.36–1.70)	<0.001
Urine protein at AKI^c^	2.25 (1.37–3.69)	0.001	1.37 (0.81–2.32)	0.24
AKI stage^d^	2.33 (1.57–3.44)	<0.001		
Surgical procedure				
Coronary artery bypass grafting^e^	1.35 (0.92–1.97)	0.13		
Valve surgery^e^	0.85 (0.57–1.28)	0.43		
Heart transplant^e^	1.47 (0.84–2.58)	0.18		
Medication				
RAS inhibitor^b^	0.47 (0.31–0.72)	<0.001	0.46 (0.30–0.70)	<0.001
Anti-HTN agents^b^	0.99 (0.76–1.29)	0.92		
Statins^b^	0.94 (0.70–1.25)	0.66		
Immunosuppressants^b^	1.19 (0.74–1.93)	0.48		

Annotation: ^a^Man compared to woman; ^b^Compared to no status; ^c^Severe compared to mild; ^d^Stage II + III compared to stage I; ^e^Compared to other cardiac surgery.

Abbreviation: CI, confidence interval; HR, hazard ratio.
